# Fatal subacute sclerosing panencephalitis in an 8-year-old male: a case report

**DOI:** 10.11604/pamj.2023.45.37.38601

**Published:** 2023-05-16

**Authors:** Fartun Abdullahi Hassan Orey, Abdirahman Omar Sahal, Bashiru Garba

**Affiliations:** 1 Department of Pediatrics and Child Health, Dr. Sumait Hospital, Faculty of Medicine and Health Sciences, SIMAD University, Mogadishu, Somalia,; 2 Sahan Diagnostic Center, Mogadishu, Somalia,; 3 Department of Public Health and Preventive Medicine, Faculty of Veterinary Medicine, Usmanu Danfodiyo University, Sokoto, Nigeria,; 4 Institute for Medical Research, SIMAD University, Mogadishu, Somalia

**Keywords:** Subacute sclerosing panencephalitis, measles, immunization, neurodegenerative disease, case report

## Abstract

Subacute sclerosing panencephalitis (SSPE) is a chronic slow progressive neurodegenerative disease that is often associated with measles complications. The disease is characterized by seizures, behavioral changes, motor deficit and eventually death. In this case report we discuss the case of an 8-year-old male who developed SSPE and was presented to our hospital with a history of generalized tonic colonic convulsion followed by gait abnormality, episodes, abnormal behaviors, and cognitive regression. On clinical exploration, the child had a history of measles at 8 months of age and meningitis at 18 months. The electroencephalogram (EEG) investigation showed high amplitude spikes, with focal seizure and slowing, while the magnetic resonance imaging reveal signals synonymous with high fluid-attenuated inversion recovery (FLAIR), both of which are consistent with probable SSPE. The case was managed symptomatically; until his parents decided to take him back home, after which his condition deteriorated, and he sadly died. To the best of our knowledge, this is the first recorded case of SSPE in Mogadishu, Somalia. Hence, the need to further investigation to better understand the incidence of the disease in the population and propose better ways of managing the condition.

## Introduction

Subacute sclerosing pan encephalitis (SSPE), is a slow progressive often fatal neurodegenerative disease caused by measles infection [1,2]. The development of SSPE follows defective measles virus maturation or reactivation in the central nervous system. The onset of the disease usually occurs in late childhood or adolescence and is generally characterized by the insidious onset of mental deterioration and myoclonia [2]. Other clinical presentations of SSPE range from dementia to abnormal jerk movement and motor dysfunction convulsion [3].

The diagnosis of SSPE can be confirmed by clinical evaluation and serology which indicate abnormally high levels of measles antibody. Other important diagnostic techniques are examination of the electrical activity of the brain (EEG) which usually shows a characteristic pattern of generalized slow-wave complexes with a regular periodicity. We report a case of an 8-year-old male who presented with a history of generalized tonic colonic convulsion followed by progressive body weakness and cognitive impairment.

## Patient and observation

**Patient information:** an 8-year-old male presented to our hospital with a history of convulsions for the last 3 months. The convulsions were focal initially but progressed to generalized tonic-clonic seizures. A high dose of sodium valproate (20mg/kg/day) was administered for control, but 3-weeks later, he developed gait abnormality, incoordinate speech, abnormal behaviors, cognitive reduction, and urine incontinence. Previously, the child was attending school, and performing well. He had histories of hospitalization at 8 months old due to measles and at 18 months due to meningitis. No history of convulsions, loss of consciousness or family history of similar illness.

**Clinical findings:** on clinical examination, the patient was stable, with no fever, hypo/ hyperpigmented lesions, or lymphadenopathy. Further examination revealed he had myoclonus ataxia, frequent spasms on extremities, with normal sensory power. Later, he developed weakness on the right side, drooling saliva and a reduced level of consciousness. He was referred to the intensive care unit.

**Diagnostics assessment:** first EEG and Magnetic resonance imaging (MRI) conducted 3 months before presentation did not show any abnormality. However, the second MRI showed high signal intensity on T2 and FLAIR-MRI in periventricular and subcortical white matter. In the third MRI, generalized reduction in the cerebral and cerebellar brain volume with hypoplastic corpus callosum and mid-lateral ventriculomegaly, non-symmetrical non-acute white matter changes was noticed, predominantly in the frontal lobe and in the mid and posterior cerebral white matter. Also, the second EEG showed focal seizure with generalized epileptic form and slowing which suggest mild encephalopathy or postictal slowing.

**Figure 1:**
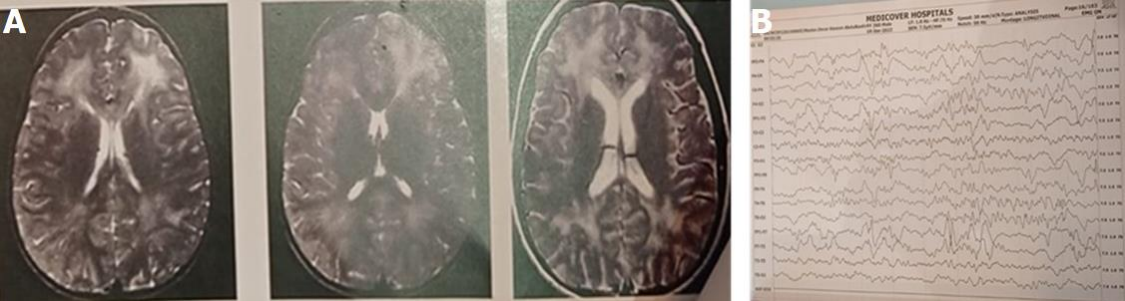
longitudinal bipolar montage showing periodic slow waves also known as the radermecker; A) Magnetic resonance imaging (MRI) demonstrating high signal intensity on T2 and FLAIR MRI in periventricular and subcortical white matter; B) EEG showing focal seizure with generalization epileptic form, suggesting encephalopathy as seen with periodic slow waves

**Therapeutic intervention:** we admitted him for 2 weeks and administered a high dose of methylprednisolone for 5 days followed by a high dose of oral prednisolone but showed no improvement. Thereafter, his family decided to take him home and he died 2 weeks later (Figure 1).

**Patient consent:** a written informed consent was obtained from the parent(s) of the patients for the publication of patient data and accompanying images.

## Discussion

SSPE is a chronic progressive disease caused by a reactivated slow measles virus (paramyxovirus) that has stayed dormant in humans for extended periods of time. In most cases, the disease occurs as a rare late complication of persistent nonproductive measles virus residing in the neurons and other parts of the central nervous system. Our patient exhibited behavioral disturbances and episodes of seizures associated with SSPE. The patient also had a history of measles disease when he was 8 months.

Available literature indicates that SSPE usually results from a measles virus infection acquired earlier in life, which in most cases develops 7 to 10 years after having the measles [4,5]. A similar pattern was also observed in our patient having recovered from measles 7 years ago. Our patient started with generalized tonic colonic convulsions, gait abnormality and abnormal behavior with an incoordinate speech that gradually progressed to lost speech. Although, early EEG and MRI scans were normal, as has been reported in previous studies. However, the condition can progress to lesions in the deep brain structures and the brainstem [6]. Similarly, as the disease progresses the EEG may give a periodic complex associated with myoclonus jerks [1]. Following the deterioration of the condition of the patient, additional EEG and MRI scans were conducted and the finding reveal focal seizure with generalized epileptic form, suggesting mild encephalopathy while EEG findings showed periodic complexes of repeated high-amplitude delta waves that are characteristics of all SSPE [7,8].

Diagnosis of SSPE is multifaceted due to the non-specific nature of the clinical manifestations. Clinicians tend to use a set of major and minor criteria for diagnosis. These include the typical presentation which can be acute, subacute, or chronic progressive/relapsing nature of the presentation and elevated anti-measles antibodies, while the atypical presentation includes seizures, among others [6]. The clinical picture of our patient conforms to the listed signs except for the absence of an antibody test. Nonetheless, we know the patient had measles in infancy.

Another minor criterion used to evaluate SSPE is EEG findings indicating high-amplitude slow waves occurring bilaterally and synchronously at fixed and regular intervals [6]. Again, the EEG of our patient was found to show these slow-wave complexes. Therefore, we conclude that the patient had SSPE. Unfortunately, he passed following the refusal of his parents to continue with the supportive treatment at the hospital.

## Conclusion

SSPE is a rare often fatal disease condition most associated with childhood measles complications. It is a neurodegenerative disease condition that results in the death of the patient due to lack of treatment. The occurrence of this case should be an eye-opener for clinicians in Somalia, especially due to the lack of advanced diagnostics and expertise within the healthcare sector. Hence, further efforts are warranted to better identify and understand the condition and its prevalence in the country.
